# IgA nephropathy: an overview of the disease, its pathophysiology, and involvement of the gut-kidney axis

**DOI:** 10.1016/j.kisu.2026.01.003

**Published:** 2026-07-20

**Authors:** Chee Kay Cheung, Laura H. Mariani

**Affiliations:** 1 John Walls Renal Unit, University Hospitals of Leicester NHS Trust, Leicester, UK; 2 Mayer IgA Nephropathy Laboratories, Department of Cardiovascular Sciences, University of Leicester, Leicester, UK; 3 Department of Internal Medicine, University of Michigan, Ann Arbor, Michigan, USA

**Keywords:** IgA, IgA nephropathy, kidney failure, pathogenesis

## Abstract

IgA nephropathy (IgAN) is the most common primary glomerular disease worldwide. Its incidence and prevalence vary widely, with estimates of annual incidence ranging from 0.06 to 10.5 per 100,000 per year in adults and children. IgAN represents an important cause of progressive kidney disease, leading to kidney failure in a large proportion of patients, and is associated with a wide spectrum of clinical symptoms, which negatively impact quality of life. Although the consequences of IgAN manifest in the kidney, multiple lines of evidence support a major role for the gut in the pathogenesis of IgAN, including genome-wide association studies that identify risk loci for IgAN that encode genes related to mucosal immunity, maintenance of the intestinal epithelial barrier, and inflammatory bowel disease. Further insights into the underlying disease pathogenesis have led to the development of a multihit model comprising 4 sequential “hits”: (i) increased levels of galactose-deficient IgA1 in the systemic circulation, (ii) followed by binding of specific autoantibodies directed against galactose-deficient IgA1, which (iii) form pathogenic IgA-containing immune complexes that (iv) deposit within the glomerular mesangium of the kidney, triggering inflammation, damage, and progressive decline in kidney function. The purpose of this article is to provide an overview of IgAN, examining its epidemiology and clinical features, and the mechanisms underlying its pathophysiology.

IgA nephropathy (IgAN) is the most common primary glomerular disease worldwide and was first described in 1968 by Jean Berger and Nicole Hinglais.^1^, ^2^, ^3^ IgAN is characterized by kidney biopsy findings of dominant or codominant IgA-containing immune deposits within the glomeruli with variable amounts of mesangial expansion and hypercellularity, endocapillary hypercellularity, and segmental glomerulosclerosis (Figure 1).^1^ These deposits lead to inflammation and kidney damage, which, in a progressive disease such as this, may lead to kidney failure within the patient’s lifetime, especially if diagnosed at a young age.^3^^,^^4^

**Key Learning Points**

• IgA nephropathy (IgAN) is an immune-mediated form of primary glomerulonephritis that is associated with a wide spectrum of clinical manifestations, which can negatively impact a patient’s quality of life and lead to progressive kidney disease in a large proportion of patients.
• Advances in the understanding of the underlying disease pathogenesis have led to the development of a multihit model whereby 4 sequential pathogenic processes or “hits” are required for IgAN to lead to kidney damage. These comprise increased levels of galactose-deficient IgA1 in the systemic circulation (hit 1), followed by binding of specific autoantibodies (hit 2), which form pathogenic IgA-containing immune complexes (hit 3) that deposit within the glomerular mesangium of the kidney (hit 4), triggering inflammation, damage, and progressive, irreversible decline in kidney function.

**Figure 1:**
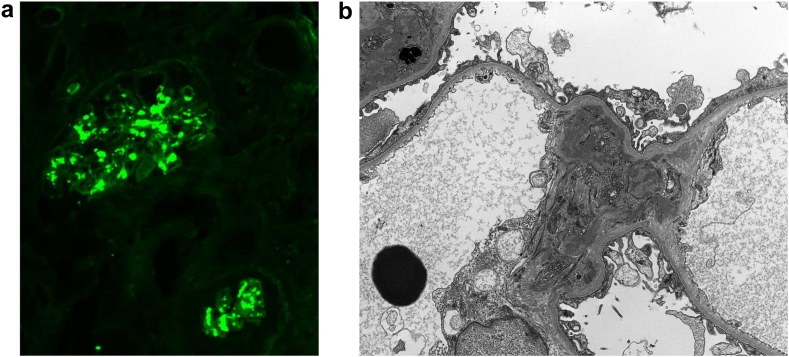
**Kidney biopsy images of IgA nephropathy.** (**a**) Immunofluorescence showing mesangial IgA deposition. (**b**) Electron microscopy showing electron-dense mesangial deposits.

The purpose of this article is to provide an overview of IgAN, including its epidemiology and clinical features, and the mechanisms underlying its pathophysiology.

## Epidemiology

Globally, estimated annual incidence rates for IgAN have been reported as ranging from 0.06 to 10.5 per 100,000 per year in adults.^5^ In children, the annual incidence of IgAN was reported to be 0.2 per 100,000 across 6 European countries compared with 9.9 per 100,000 in Japan. The higher incidence in Japan may be explained by the presence of a nationwide urinalysis screening program for school-aged children, leading to higher detection rates, as well as differences in the underlying disease incidence.^6^^,^^7^ A higher disease incidence is noted in Asian versus White populations, and the lowest incidence is reported among African populations.^8^ Although genetic factors are likely to explain much of this variability, this may also be partly attributable to differences in health care access, clinical practice (e.g., urinalysis screening and clinical thresholds to perform a kidney biopsy), and completeness of data records that make it difficult to accurately assess the incidence of IgAN across populations.^3^^,^^9^, ^10^, ^11^ Variation in gender distribution by race has been reported, with a male/female ratio of ∼1:1 in Asian populations and ∼2 to 3:1 in White populations.^3^^,^^8^^,^^9^

Genome-wide association studies (GWASs) have identified over 30 risk loci associated with IgAN that account for 11% of disease risk.^12^ A high polygenic risk score is associated with earlier onset of IgAN and an increased lifetime risk of kidney failure.^12^ The frequency of risk loci involved in antigen presentation, mucosal immunity, complement receptor formation, and alternative complement pathway activation was found to be higher in Han Chinese populations compared with European populations, which may explain the more severe clinical presentations and higher risk of disease progression in Chinese patients.

## Clinical Presentation and Diagnosis

**Figure 2:**
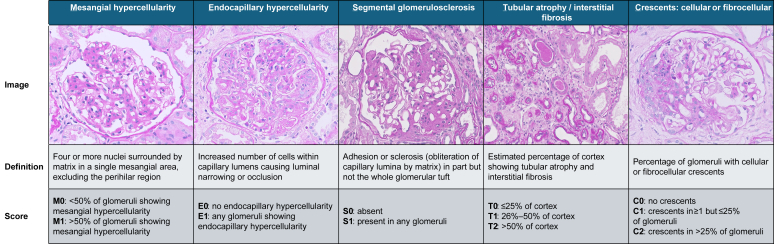
**MEST-C (mesangial hypercellularity, endocapillary hypercellularity, segmental glomerulosclerosis, tubular atrophy/interstitial fibrosis, and crescents) criteria in the Oxford classification of IgA nephropathy.**^18^, ^19^, ^20^, ^21^ Adapted from the publications of the Working Group of the International IgA Nephropathy Network and the Renal Pathology Society.^18^, ^19^, ^20^, ^21^ The histology images are reproduced with kind permission from the copyright holder, Ian Roberts, Professor of Cellular Pathology, Oxford University Hospitals, Oxford, UK.

IgAN is a highly variable disease that is associated with a range of clinical signs and symptoms.^13^ The most common clinical manifestations include proteinuria, decline in kidney function, nonvisible or visible hematuria (occasionally following an upper respiratory tract infection or gastrointestinal tract illness), and hypertension.^3^^,^^13^ Rarely, patients may present with rapidly progressive glomerulonephritis and acute kidney injury or with nephrotic syndrome.^13^ In clinical practice, proteinuria and estimated glomerular filtration rate (eGFR) are used to stratify the risk of disease progression, although changes in these parameters may only be detected after irreversible kidney damage has occurred.^3^^,^^4^^,^^14^^,^^15^ A kidney biopsy is required to diagnose IgAN, and currently there are no validated serum or urine biomarkers that can be used for diagnosis.^3^^,^^13^ Potential biomarkers and their role in IgAN are discussed in detail in the article “The expanding role of biomarkers in the management of IgA nephropathy” by Jain and Rizk^16^ of this supplement. It is recommended that the kidney biopsy is graded according to the Oxford classification, which includes the following histologic components: mesangial (M) and endocapillary (E) hypercellularity, segmental glomerulosclerosis (S), tubular atrophy/interstitial fibrosis (T), and crescents (C)^17^, ^18^, ^19^, ^20^, ^21^, ^22^ (Figure 2).

**Figure 3:**
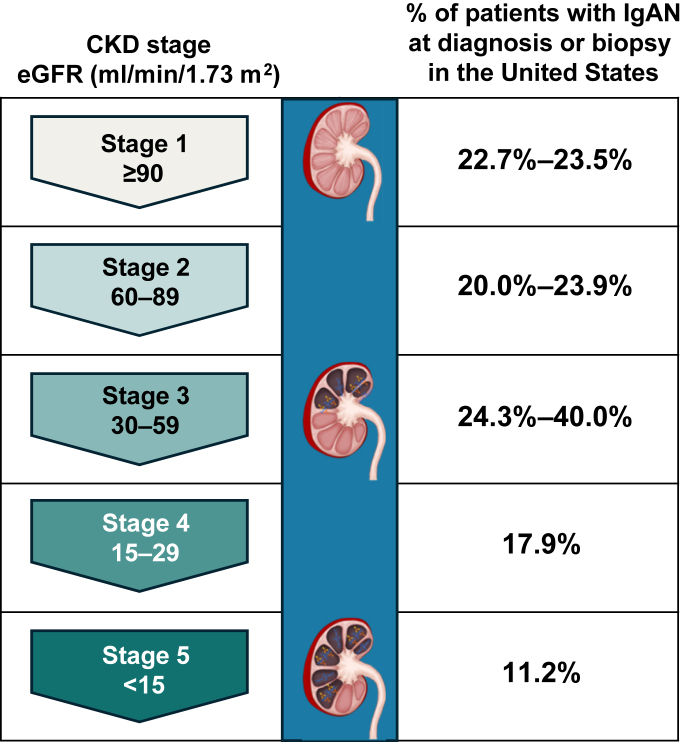
**Distribution of patients with IgA nephropathy (IgAN) across the different chronic kidney disease (CKD) stages at diagnosis or biopsy in the United States.**^24^^,^^25^ eGFR, estimated glomerular filtration rate.

Given the higher incidence of IgAN in East Asian populations, nationwide mass urinary screening programs have been instituted in Japan, South Korea, and Taiwan, allowing for the earlier identification of kidney disease and the potential for early intervention.^7^^,^^9^ The absence of screening programs in other countries is likely to impact the stage at which IgAN is diagnosed.^9^ A recent survey reported the frequency of preserved kidney function (defined as eGFR >60 ml/min per 1.73 m^2^) at the time of kidney biopsy to be lower in Europe (55%) compared with Japan (71%).^23^ Similarly in the United States, a high proportion of patients present with chronic kidney disease stage ≥3 (Figure 3).^24^^,^^25^

In most countries, patients with IgAN are diagnosed in early to mid-adult life with reduced kidney function, indicating that significant nephron loss has already occurred by the time of their diagnosis.^4^^,^^26^ Data from the UK National Registry of Rare Kidney Diseases (RaDaR) IgAN cohort that included 2299 adults and 140 children with biopsy-proven IgAN and proteinuria >0.5 g/d or eGFR <60 ml/min per 1.73 m^2^ demonstrated that most of the cohort progressed to kidney failure within 10 to 15 years. Even those traditionally considered as “low-risk” patients (with proteinuria <1 g/d) were at increased risk of progressing to kidney failure within their lifetime.^4^ These findings are supported by data from long-term observational registry studies from Sweden and China, and the European Validation Study of the Oxford Classification of IgAN (VALIGA) cohort.^27^, ^28^, ^29^ According to epidemiological studies, the diagnosis of IgAN was associated with a reduction in life expectancy that was mainly attributable to complications of kidney failure, by a median of 6 and 10 years in Sweden and the United States, respectively.^30^^,^^31^ Recurrent IgAN after kidney transplantation is common, and approximately 50% to 60% of patients develop evidence of histologic recurrence within 5 years,^32^ which is associated with reduced long-term graft survival.

The International IgAN Prediction Tool can be used to predict the risk of kidney disease progression. Both clinical and histologic parameters (from the Oxford classification) at the time of kidney biopsy, or up to 2 years after biopsy, are used to calculate the risk of a 50% reduction in eGFR or kidney failure (eGFR <15 ml/min per 1.73 m^2^) up to 7 years from diagnosis in adults and children.^33^, ^34^, ^35^, ^36^, ^37^, ^38^ This tool is also discussed in the article “Management of IgA nephropathy and the expanding role of immunomodulation” by Canetta and Reich^39^ of this supplement.

## Pediatric IGAN

IgAN is less frequently diagnosed in children than in adults in European countries. According to European registries, the annual incidence of IgAN was estimated at 0.76 per 100,000 in patients of all ages, 0.2 per 100,000 in children, and 0.3 per 100,000 in elderly patients.^6^ In countries where screening programs for school-aged children exist (e.g., Japan), IgAN is detected more frequently.^40^ In pediatric patients with IgAN, hematuria is more frequently observed and patients have a higher eGFR at diagnosis.^41^, ^42^, ^43^ Active lesions, such as mesangial proliferation, are more commonly observed on the initial diagnostic kidney biopsy than in adults, whereas chronic lesions, such as tubular atrophy and interstitial fibrosis, are less frequently reported.^41^^,^^42^ The International IgAN Prediction Tool has been adapted for use in pediatric patients to predict a 30% decline in eGFR, or end-stage kidney disease.^43^^,^^44^ The Oxford classification of IgAN has also been validated in children.^45^ The pediatric cohort of the UK RaDaR study demonstrated a median time to first event (kidney failure or death) of over double that of the adult cohort (10.2 vs. 4.3 years) and significantly longer kidney survival (*P* < 0.001).^4^ However, outcomes for both groups remained poor, and for those diagnosed as children, the mean age at developing kidney failure was 27 years.^4^

## Pathogenesis of IGAN

IgAN is an immune-mediated disease influenced by an interplay between genetic and environmental factors that is not completely understood. As mentioned, GWASs have identified over 30 different risk loci for IgAN to date; environmental factors may include infections or food antigens, potentially leading to alterations in host microbiota and aberrant mucosal immune responses.^3^^,^^12^^,^^46^, ^47^, ^48^, ^49^, ^50^

### The gut-kidney axis in IgAN

Although IgAN manifests in the kidney, there is strong evidence to support the role of the gut in the pathogenesis of IgAN.^51^ The concept of a “gut-kidney” axis has been proposed based on several observations, including gut mucosal infections leading to “flares” of IgAN with visible hematuria in certain patients, as well as differences in gut microbiota composition between healthy individuals and patients with IgAN.^51^^,^^52^ Pediatric patients with IgAN more frequently present with visible hematuria after mucosal infections (e.g., upper respiratory tract or gastrointestinal tract infections) than adults.^41^^,^^53^, ^54^, ^55^

#### Genetic factors

Risk loci for IgAN identified via GWASs are also associated with other gastrointestinal tract diseases, including Crohn’s disease and ulcerative colitis.^12^^,^^46^ A Swedish population-based cohort study reported that patients with IgAN had a higher risk of developing inflammatory bowel disease compared with matched controls. Among patients with IgAN, comorbid inflammatory bowel disease increased the risk of progression to kidney failure.^56^

Analysis of risk loci identified by GWAS highlighted the intestinal immune network for IgA production to be a major enriched pathway in patients with IgAN.^12^^,^^46^^,^^62^ GWASs also identified *DEFA* and *TNFSF13* as risk loci, which encode proteins (α-defensins and a proliferation-inducing ligand [APRIL], respectively) implicated in the mucosal immune response to intestinal antigens.^46^

Noncoding RNAs may also contribute toward the pathogenesis of IgAN. For example, microRNAs that regulate the expression of enzymes involved in IgA1 *O*-glycosylation (miR-148b, miR-374b, and let-7b) or have a potential role in kidney fibrosis (miR-204) have been shown to be differentially expressed in patients with IgAN compared with those without the disease.^3^^,^^57^, ^58^, ^59^, ^60^

#### Mucosal immune dysregulation

**Figure 4:**
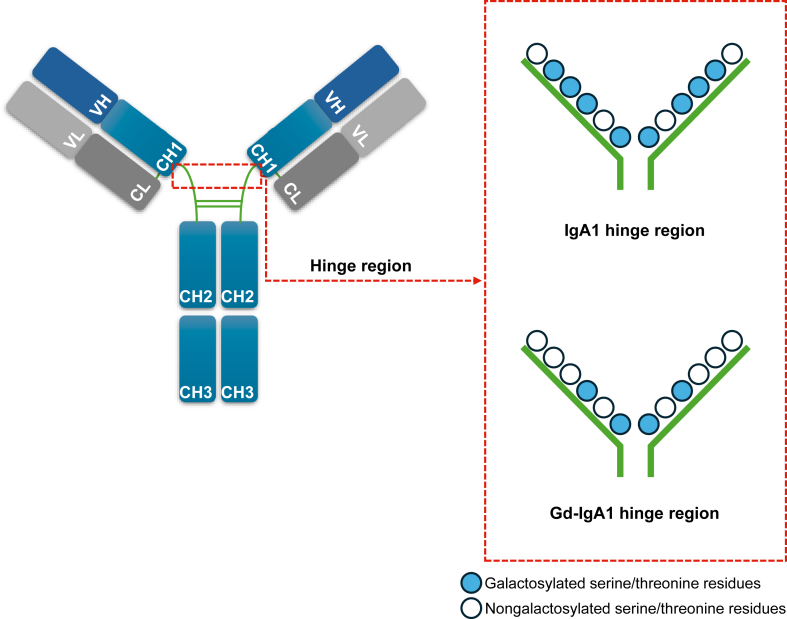
**IgA1 structure and differences between glycosylated (top-right) and poorly glycosylated (bottom-right) forms.**^3^^,^^63^^,^^64^ IgA1 contains a hinge region with serine or threonine residues that undergo variable glycosylation through the enzymatic addition of carbohydrate side chains. IgA1 that lacks terminal galactose from these side chains, resulting in poorly galactosylated IgA1 glycoforms, is termed galactose-deficient IgA1 (Gd-IgA1), and is present in higher concentrations in the circulation and within glomerular deposits in IgA nephropathy. C, constant region; H, heavy chain; L, light chain; V, variable region.

IgA plays a key role as part of the first line of host defense at gut mucosal surfaces, where it prevents the attachment of pathogens to epithelial cells and traps them in the mucus layer, ready for excretion in feces.^3^^,^^51^ IgA secreted into the intestinal lumen can also influence inflammatory responses, transporting antigens to immune cells in the gut-associated lymphoid tissue (GALT) via specialized endothelial cells (M cells).^3^ Humans (and higher primates) possess 2 IgA isoforms, IgA1 and IgA2, which differ in that IgA1 includes an extended hinge region that is absent in IgA2.^61^^,^^62^ The IgA1 hinge region undergoes posttranslational *O*-glycosylation, with the variable addition of oligosaccharide side chains (Figure 4).^3^^,^^61^^,^^63^^,^^64^ Circulating IgA1 glycoforms that lack terminal galactose from these IgA1 hinge region side chains, referred to collectively as galactose-deficient IgA1 (Gd-IgA1), are found at elevated levels in patients with IgAN compared with healthy individuals.^3^

The presence of elevated levels of Gd-IgA1 alone does not necessarily translate into kidney injury, because higher levels may also be detected in asymptomatic relatives of patients with IgAN who display no evidence of kidney disease.^65^ Gd-IgA1 is considered to be the “pathogenic” form of IgA in IgAN, because of its tendency to self-aggregate, bind to serum proteins, and be recognized by specific IgA and IgG autoantibodies to form immune complexes.^3^^,^^61^^,^^66^ Gd-IgA1 circulating immune complexes may also contain cluster of differentiation 89 (CD89), an Fc receptor for IgA. Binding of IgA1 to myeloid cells may lead to shedding of CD89, generating soluble CD89 that contributes to circulating immune complex formation, and has been proposed to contribute to the disease pathogenesis.^67^

Pathogenic IgA1 is thought to be produced by B cells that originate from the mucosal-associated lymphoid tissue (MALT).^3^^,^^51^^,^^52^^,^^68^ IgA1 found within glomerular deposits in patients with IgAN is predominantly polymeric, as is IgA produced by mucosal effector sites.^69^ Of all the MALT sites, the GALT produces the most IgA, estimated to be between 3 and 5 g/d (the majority of the body’s total Ig production), which is secreted into the intestinal lumen.^3^^,^^51^ A major component of the GALT is the Peyer patches of the small intestine, which consist of aggregated lymphoid follicles rich in B cells; almost half of the Peyer patches are located in the distal part of the ileum.^51^^,^^70^ In certain patients, the nasal-associated lymphoid tissue (NALT), including the tonsils, may also be a source of pathogenic IgA1, with upper respiratory tract infections being a trigger for flares of nephritis and visible hematuria. Tonsillectomy is used as a treatment option in Japan to debulk the NALT, but evidence for its effectiveness is lacking in other populations.^51^^,^^52^^,^^71^^,^^72^

In Peyer patches, exposure to antigens from the intestinal lumen induces a class switch in naïve B cells (which express IgM and IgD) to IgA-producing B cells. This occurs via both T-cell–dependent and T-cell–independent pathways.^73^ The T-cell–dependent pathway includes toll-like receptor (TLR) ligand recognition on dendritic cells, which promotes differentiation of naïve T cells into follicular helper T cells; these cells then promote B-cell IgA class switching. T-cell–independent induction of IgA-producing B cells occurs mainly through interactions with the cytokines B-cell–activating factor (BAFF) and APRIL, which are secreted by myeloid cells (including activated dendritic cells, macrophages, and monocytes, as well as epithelial cells and T cells) in the lamina propria of the intestinal mucosa.^72^, ^73^, ^74^, ^75^, ^76^, ^77^, ^78^ Elevated serum levels of both BAFF and APRIL have been reported in patients with IgAN, and correlate with more severe histologic activity on kidney biopsy, increased proteinuria, and worse kidney outcomes.^79^, ^80^, ^81^, ^82^ Therefore, Peyer patches function as key immune priming sites in which B cells are activated by gut-derived antigens, leading to the proliferation and expansion of antigen-specific IgA-producing mucosal B cells.^73^, ^74^, ^75^ IgA1 derived from mucosally primed B cells is more likely to be poorly *O*-galactosylated (i.e., galactose deficient) than that from systemically primed B cells.^83^^,^^84^

#### Environmental factors

Alterations in gut microbiota and the intestinal epithelial barrier have been identified as potential modulators of IgA glycosylation and mucosal immune responses in IgAN. Dysbiosis and barrier dysfunction can increase exposure of mucosal immune cells within Peyer patches to microbial products, such as lipopolysaccharides, which activate TLR4 pathways.^67^^,^^85^^,^^86^ TLR4 expression has been shown to be elevated in patients with IgAN and correlates with markers of disease activity.^67^^,^^85^ Activation of TLR4 in dendritic cells leads to increased expression of BAFF and APRIL, which promote T-cell–independent class switching of naïve B cells to IgA-producing cells, and *in vitro* influence the activity of glycosyltransferases involved in hinge region galactosylation.^67^^,^^85^ Sustained or excessive TLR stimulation may, therefore, increase production of IgA and Gd-IgA1. Additionally, specific gut microbes or their enzymes may directly modify secretory IgA molecules, potentially generating neoepitopes that form immune complexes.^85^^,^^86^ Together, these processes suggest that microbial and barrier-related immune responses may increase Gd-IgA1.^67^^,^^85^^,^^86^

A gut source of pathogenic IgA in IgAN is also supported by evidence from murine models. In a transgenic mouse model that expresses human IgA1, depletion of gut microbiota by broad-spectrum antibiotics abrogated the development of spontaneous IgAN, with reductions in circulating human IgA1-IgG immune complexes, human IgA1 mesangial deposition, glomerular inflammation, and proteinuria.^87^ Treatment with antibiotics could also reverse established disease.^87^ Furthermore, fecal microbiota transplantation from either patients with progressive IgAN or healthy participants influenced the severity of disease in the model.^88^ Transgenic mice overexpressing BAFF exhibit IgAN-like pathology, including elevated serum IgA levels and glomerular IgA deposits.^89^ Glomerular IgA deposition did not occur if transgenic mice overexpressing BAFF were raised in a germ-free environment, but occurred once gut bacterial flora were introduced.^89^ Cross-sectional studies in patients with IgAN and healthy participants identified differences in gut microbiota composition, with a higher proportion of *Bacteroides*, *Escherichia-Shigella*, and *Ruminococcus* species observed in patients with IgAN.^90^ IgAN disease severity might also be associated with gut microbiota; patients with hematuria or raised proteinuria levels had higher *Escherichia-Shigella* and lower *Bifidobacterium* levels.^90^^,^^91^

As opposed to human and higher primate IgA isoforms, murine IgA more closely resembles human IgA2, as it lacks an extended hinge region; therefore, the ability of mouse models to encompass all aspects of human IgAN is limited. Marmosets have been studied in this regard and, notably, in captivity, were found to be prone to developing intestinal issues and glomerulopathies that are similar to human IgAN, which has been attributed to a change in their diet. In a murine model of IgAN that expresses human IgA1 and CD89, introduction of a gluten-containing diet increased intestinal IgA1 secretion and serum IgA1 anti-gliadin antibodies.^92^

The mechanism by which pathogenic Gd-IgA1 from a mucosal source enters the circulation in IgAN has not been fully elucidated. Possible mechanisms include overspill of mucosal IgA into the circulation, or mistrafficking of mucosally primed IgA^+^ B cells to systemic sites, such as the bone marrow, or even the kidneys themselves.^3^^,^^93^, ^94^, ^95^, ^96^, ^97^ A recent study demonstrated a higher relative abundance of mucin-degrading bacteria (including *Akkermansia muciniphila*) within the gut microbiota of patients with IgAN compared with individuals with other forms of chronic kidney disease. These bacteria were demonstrated to deglycosylate IgA1 *in vitro*, and in the gut lumen in murine models, which led to retrotranscytosis (reverse trafficking) into the circulation.^98^ An earlier murine study suggested that dysregulated LIGHT expression (a ligand for lymphotoxin β receptor [LTβR], encoded by *TNFSF14*) could lead to defective IgA transportation into the gut lumen and increases in polymeric IgA in the serum, leading to features similar to IgAN.^3^^,^^99^

Taken together, the gut appears to play an important role in the pathogenesis of IgAN and the initial generation of pathogenic IgA^+^ B cells.^100^ Furthermore, a targeted-release formulation of budesonide, Nefecon, which is designed to be released at the distal ileum and have an immunomodulatory effect on Peyer patches at the gut mucosal level, was clinically effective in terms of reducing proteinuria and preserving kidney function in a large phase 3 clinical trial.^100^, ^101^, ^102^ This is discussed in more detail in the article titled “Approved therapies in the IgA nephropathy armamentarium: a summary of the evidence” by Norouzi and Lafayette^103^ in this supplement.

### The multihit model of IgAN

**Figure 5:**
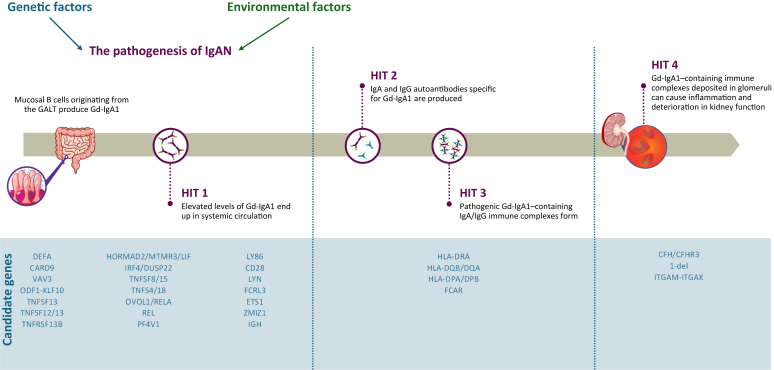
**Multihit hypothesis of IgA nephropathy (IgAN) pathogenesis and the potential candidate genes involved, based on genome-wide association study (GWAS) findings.**^12^^,^^47^^,^^51^^,^^52^^,^^104^, ^105^, ^106^, ^107^ GALT, gut-associated lymphoid tissue; Gd-IgA1, galactose-deficient IgA1.

A multihit hypothesis for IgAN pathogenesis, supported by GWASs, is widely accepted, where 4 sequential pathogenic processes or “hits” are required (Figure 5).^12^^,^^47^^,^^51^^,^^52^^,^^104^, ^105^, ^106^, ^107^ First, production and accumulation of Gd-IgA1 are observed in the systemic circulation, as discussed above (hit 1).^104^

Second, IgA and IgG antibodies are produced, which are specific to exposed N-acetylgalactosamine (GalNAc) residues at the hinge region of Gd-IgA1 (hit 2).^104^^,^^108^ Whether these are true autoantibodies that are generated because of a loss of tolerance to increased levels of Gd-IgA1 in the circulation, cross-reactive antibodies that are initially formed against GalNAc motifs on bacterial cell walls, or a combination of these processes is not clear and may differ between individuals. Notably, serum levels of IgG antibodies specific for Gd-IgA1 were shown to correlate with disease severity, in terms of magnitude of proteinuria and long-term kidney outcomes.^104^^,^^108^^,^^109^

Third, binding of Gd-IgA1 to circulating IgA/IgG antibodies leads to the formation of Gd-IgA1–IgA/IgG immune complexes (IgA1-IgA/IgG-IC) (hit 3).^3^^,^^104^^,^^108^ High levels of circulating IgA1-IgG-IC have been noted in patients with IgAN and were found to increase during disease activity, in terms of episodes of visible hematuria.^3^^,^^110^, ^111^, ^112^

Last, these circulating immune complexes, because of their size and composition, are not cleared by the liver, but have a propensity to deposit within the glomerular mesangium of the kidney (hit 4).^104^^,^^113^ This accumulation of IgA immune complexes can result (to varying extents, depending on an individual’s susceptibility) in mesangial cell activation, proliferation, recruitment of inflammatory cells, secretion of cytokines and extracellular matrix components, and complement activation.^31^^,^^104^^,^^113^, ^114^, ^115^ These events can cumulatively lead to podocyte injury, affecting their structure and function, and disruption of the glomerular filtration barrier, which lead to the clinicopathologic characteristics of IgAN, including hematuria, proteinuria, tubulointerstitial inflammation, and fibrosis, and eventually the progressive and permanent loss of kidney function.^3^^,^^4^^,^^13^^,^^32^^,^^104^^,^^115^^,^^116^ Because fibrosis is a common manifestation of chronic kidney disease, shared fibrotic mechanisms between IgAN and other kidney diseases are likely to exist.^117^

Circulating IgA immune complexes in IgAN contain other bioactive components, including complement components, which may contribute toward complement activation, accelerating glomerular inflammation and kidney damage.^3^^,^^48^^,^^113^, ^114^, ^115^^,^^118^ The complement system is activated via 3 pathways: the classical, lectin, and alternative pathways, which all converge on the terminal pathway.^113^^,^^119^^,^^120^ The alternative pathway is thought to play a major role in complement activation in IgAN and is linked to C3 deposition.^113^^,^^119^ Factor B– and factor H–related proteins that promote alternative pathway activity have been identified in kidney biopsies of patients with IgAN, and their presence was associated with progressive disease.^119^^,^^121^, ^122^, ^123^, ^124^, ^125^ Alternative complement breakdown products have also been detected within circulating immune complexes and colocalized with IgA glomerular deposits.^119^^,^^121^ Interactions between IgA and the lectin pathway act as important mediators of innate immunity in the respiratory and gastrointestinal tracts. Lectin pathway components, including C4d, mannose-binding lectin, and mannose-binding lectin–associated serine proteases 1 and 2, have also been observed within glomerular deposits in IgAN, and their presence is associated with worse long-term prognosis.^123^^,^^126^ C4d glomerular deposition was detected in 39% and 25% of patients in 2 large case series from Spain and the Netherlands, respectively, and its presence was associated with worse kidney histologic findings, a more rapid decline in eGFR, and a higher risk of kidney failure.^113^^,^^123^^,^^126^, ^127^, ^128^, ^129^ C1q deposition is absent in most cases of IgAN, suggesting that the classical pathway of the complement system does not play a major role in IgAN pathogenesis.^104^^,^^115^ When detected, the presence of C1q was associated with worse outcomes in cohorts from Asia.^125^^,^^130^, ^131^, ^132^ The role of the complement pathway in the pathogenesis of IgAN is discussed further in the article “The expanding role of biomarkers in the management of IgA nephropathy” by Jain and Rizk^16^ of this supplement.

Following the glomerular deposition of IgA immune complexes, several downstream pathways are activated, including the renin-angiotensin and endothelin systems, promoting deleterious hemodynamic and intraglomerular effects.^3^^,^^48^^,^^133^ Originally identified as an endothelium-derived vasoconstrictor, endothelin-1 (ET-1) is a 21–amino acid peptide that is now known to elicit a range of responses across multiple organs and tissues.^48^ ET-1 is produced in large amounts in the kidney, and the endothelin A and B receptors are expressed by several kidney cell types.^48^ ET-1 plays an important role in regulating renal blood flow and maintaining homeostasis, and its production in the kidney is stimulated by (among others) inflammatory, fibrotic, vasoactive, proliferative, and oxidative processes.^48^^,^^134^^,^^135^ Kidney ET-1 expression has previously been shown to be increased in IgAN.^136^ Although endothelin B receptor activation in the kidney leads to vasodilation and natriuresis, the binding of ET-1 to endothelin A receptor triggers a range of effects that promote disease progression in IgAN, including vasoconstriction and glomerular hyperpermeability, mesangial cell activation, podocyte injury, glomerular and tubulointerstitial inflammation, and fibrosis.^48^^,^^134^^,^^136^ Endothelin A receptor activation within the kidney has also been associated with worsening proteinuria and reduced kidney function in patients with IgAN. Furthermore, ET-1 can be produced via positive feedback pathways that result from these processes; for example, the exposure of renal tubular cells to urinary protein induces them to produce more ET-1. This, in turn, contributes to more tubulointerstitial damage.^48^^,^^137^ ET-1, therefore, has a role in enacting kidney damage in IgAN.

## Summary and Conclusions

Our understanding of the underlying disease pathogenesis in IgAN has advanced significantly over the past decades, and cumulative data have led to the development of a multihit model. IgAN is recognized as a disease that exhibits marked heterogeneity between individuals and ethnic groups, in terms of its clinical and pathologic features, its epidemiology, and rate of disease progression, with long-term outcomes differing greatly across global populations. The reasons behind this heterogeneity are not yet fully understood, but genetic factors are likely to play an important role.^12^

Given that a significant loss of nephrons has often already occurred by the time of diagnosis and the high likelihood of a young adult patient presenting with IgAN progressing to kidney failure during his/her lifetime, there is an urgent need to diagnose and treat patients as early as possible in their disease course to provide optimal outcomes, in terms of preserving remaining nephron function, delaying or preventing kidney failure, and maintaining quality of life. Further work is required, especially on the development of reliable noninvasive diagnostic and prognostic biomarkers, to enable optimal individualized treatment and ensure that patients receive the right treatment at the right time. Our improved understanding of the pathogenesis of IgAN has allowed for the development of several novel therapeutic strategies that are targeted to specific areas of the multihit model and the downstream pathways that are activated following the glomerular deposition of pathogenic IgA immune complexes and nephron loss. The following articles in this supplement explore these elements in more detail (“Management of IgA nephropathy and the expanding role of immunomodulation,” by Canetta and Reich^39^, “Approved therapies in the IgA nephropathy armamentarium: a summary of the evidence,” by Norouzi and Lafayette^103^, and “IgA nephropathy management: what does the future hold?” by Tang and Reich^138^).

## Disclosure

This article is published as part of a supplement sponsored by Calliditas Therapeutics, an Asahi Kasei company.

CKC reports receiving consulting fees from Alexion, Boehringer Ingelheim, CSL Vifor, Emerald Clinical, Novartis, Otsuka Pharmaceutical, STADA, Travere Therapeutics, Vera Therapeutics, and Vertex; receiving speaker fees from Calliditas Therapeutics, CSL Vifor, Novartis, Otsuka Pharmaceutical, STADA, Travere Therapeutics, Vera Therapeutics, and Vertex; receiving grant support to his institution from Travere Therapeutics; being on a data monitoring committee for Roche; and having an advisory or leadership role as treasurer of the International IgA Nephropathy Network. LHM reports receiving consulting fees from Calliditas Therapeutics, Dimerix, Novartis-Chinook Therapeutics, Travere Therapeutics, and Vera Therapeutics; and receiving grant support to her institution from Boehringer Ingelheim, Calliditas Therapeutics, HI-Bio, NephCure Kidney International, Reliant Glycosciences, Takeda Therapeutics, and Travere Therapeutics.
